# *Bacteroides thetaiotaomicron* and *Faecalibacterium prausnitzii* influence the production of mucus glycans and the development of goblet cells in the colonic epithelium of a gnotobiotic model rodent

**DOI:** 10.1186/1741-7007-11-61

**Published:** 2013-05-21

**Authors:** Laura Wrzosek, Sylvie Miquel, Marie-Louise Noordine, Stephan Bouet, Marie Joncquel Chevalier-Curt, Véronique Robert, Catherine Philippe, Chantal Bridonneau, Claire Cherbuy, Catherine Robbe-Masselot, Philippe Langella, Muriel Thomas

**Affiliations:** 1 INRA, UMR 1319 MICALIS, AgroParisTech, Domaine de Vilvert, Jouy-en-Josas, 78350, France; 2 AgroParisTech, UMR 1319 MICALIS, Jouy-en-Josas, F-78350, France; 3 Commensal and Probiotics-Host Interactions Laboratory, Jouy-en-Josas, F-78350, France; 4 INRA, UMR 1313, Animal Genetics and Integrative Biology, Jouy-en-Josas, F-78350, France; 5 Laboratory of Radiobiology and Genomics Studies, CEA, DSV, IRCM, SREIT, Jouy-en-Josas, F-78350, France; 6 AgroParisTech, UMR 1313, Animal Genetics and Integrative Biology, Jouy-en-Josas, F-78350, France; 7 Univ Lille Nord de France, Lille, F-59000, France; 8 USTL, UGSF, IFR 147, CNRS, Villeneuve d’Ascq, F-59650, France; 9 UMR 8576, Villeneuve d’Ascq, F-59650, France; 10 Amipem Team, Jouy-en-Josas, F-78350, France

**Keywords:** Germ-free rats, Goblet cells, Mucin O-glycosylation, KLF4, Short chain fatty acid

## Abstract

**Background:**

The intestinal mucus layer plays a key role in the maintenance of host-microbiota homeostasis. To document the crosstalk between the host and microbiota, we used gnotobiotic models to study the influence of two major commensal bacteria, *Bacteroides thetaiotaomicron* and *Faecalibacterium prausnitzii,* on this intestinal mucus layer. *B. thetaiotaomicron* is known to use polysaccharides from mucus, but its effect on goblet cells has not been addressed so far. *F. prausnitzii* is of particular physiological importance because it can be considered as a sensor and a marker of human health. We determined whether *B. thetaiotaomicron* affected goblet cell differentiation, mucin synthesis and glycosylation in the colonic epithelium. We then investigated how *F. prausnitzii* influenced the colonic epithelial responses to *B. thetaiotaomicron*.

**Results:**

*B. thetaiotaomicron*, an acetate producer, increased goblet cell differentiation, expression of mucus-related genes and the ratio of sialylated to sulfated mucins in mono-associated rats. *B. thetaiotaomicron,* therefore, stimulates the secretory lineage, favoring mucus production. When *B. thetaiotaomicron* was associated with *F. prausnitzii*, an acetate consumer and a butyrate producer, the effects on goblet cells and mucin glycosylation were diminished. *F. prausnitzii*, by attenuating the effects of *B. thetaiotaomicron* on mucus, may help the epithelium to maintain appropriate proportions of different cell types of the secretory lineage. Using a mucus-producing cell line, we showed that acetate up-regulated KLF4, a transcription factor involved in goblet cell differentiation.

**Conclusions:**

*B. thetaiotaomicron* and *F. prausnitzii*, which are metabolically complementary, modulate, *in vivo*, the intestinal mucus barrier by modifying goblet cells and mucin glycosylation. Our study reveals the importance of the balance between two main commensal bacteria in maintaining colonic epithelial homeostasis via their respective effects on mucus.

## Background

Understanding the crosstalk between the microbiota and the intestinal epithelium is essential because of the involvement of the intestinal microbiota in human health [1]. About 90% of the bacterial community that composes the intestinal microbiota and colonizes the gastrointestinal tract (GIT) are members of the phyla Bacteroidetes or Firmicutes [2-4]. *B. thetaiotaomicron* is a Gram negative bacterium, belonging to the *Bacteroides* genus from the Bacteroidetes phylum. *Faecalibacterium prausnitzii* is a Gram positive bacterium, in the *Clostridium leptum* group from the Firmicutes phylum. These two species are metabolically complementary: *B. thetaiotaomicron* is an acetate producer whereas *F. praunistzii* is an acetate consumer and a butyrate producer [5,6]. Both are major components of the intestinal microbiota and, therefore, contribute to the interactions between the microbiota and the GIT. To establish a relevant simplified model of microbiota, we studied these two commensal bacteria as functional members of the microbiome influencing host metabolism [7].

The intestinal mucus layer is located at the interface between the intestinal epithelium and the microbiota; it is, therefore, a key factor in the crosstalk between the intestinal epithelium and the microbiota and, hence, in the maintenance of intestinal homeostasis. Disruption of this protective layer may lead to inflammation [8]. Mucus is continuously secreted into the GIT lumen by specialized epithelial cells, the goblet cells, and is mainly composed of heavily O-glycosylated proteins called mucins [9]. The number and function of goblet cells are modulated by the microbiota [10-12]. However, neither the species nor the mechanisms involved in this process have been described. The microbiota expresses a wide range of carbohydrate-degrading enzymes which process otherwise indigestible dietary compounds and mucus polysaccharides [13,14]. The host genome does not encode homologs of many of these enzymes, evidence of the complementarity between host and microbiota and illustrating the adaptation of the intestinal microbiota to the host digestive environment [15,16]. *B. thetaiotaomicron* possesses a large repertoire of genes involved in sensing and hydrolyzing numerous diet- and host-derived polysaccharides [17,18]. According to nutrient availability, *B. thetaiotaomicron* can redirect its metabolism from dietary polysaccharides to host-derived polysaccharides, including mucus, and *vice versa*, and further refine its niche specificity [19,20]. This ability of *B. thetaiotaomicron* to grow on mucus contributes to its colonization and persistence in the GIT [18]. Although the ability of *B. thetaiotaomicron* to use polysaccharides from mucus has been described, little is known about its effect on goblet cells.

*F. prausnitzii* has been only poorly described *in vivo*; it is extremely oxygen sensitive and thus difficult to cultivate. Nevertheless, various observations link *F. prausnitzii* with health status. *F. prausnitzii* is of particular physiological importance because it can be considered as a sensor and a marker of human health [21]. Intestinal disorders, such as inflammatory bowel diseases [22-25], irritable bowel syndrome [26], colorectal cancer [27,28] and obesity [29] have all been reported to be associated with a diminished prevalence and abundance of *F. prausnitzii*. Moreover, several diseases have been shown to be associated with abnormal Bacteroidetes to Firmicutes ratios [1]. Therefore, analysis of the species *B. thetaiotaomicron* and *F. prausnitzii* may be informative about the crosstalk between the host and microbiota. The first objective of our work was to determine whether *B. thetaiotaomicron* affects goblet cell differentiation, mucin synthesis and glycosylation in the colonic epithelium. We then investigated how *F. prausnitzii* influences the colonic epithelial responses to *B. thetaiotaomicron*. For this study, we developed a new model based on *B. thetaiotaomicron* and *F. prausnitzii* di-associated gnotobiotic rodents to show metabolic cooperation between these species. This work illustrates how two bacteria, representative of the two main phyla, can modulate the mucus profile in the colon.

## Results

### Establishment of a model of *Bacteroides thetaiotaomicron* mono-associated rats stable as early as 2 days and up to 30 days after inoculation

**Figure 1:**
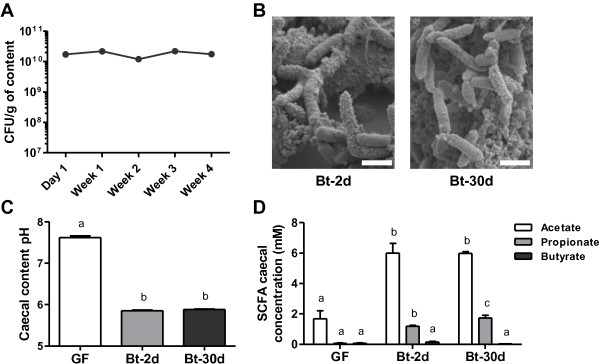
**Time course analysis of GIT in *B. thetaiotaomicron *mono-associated rats.** Germfree (GF) rats were inoculated with a culture of *B. thetaiotaomicron* (10^7^ CFU) and euthanized 2 days (Bt-2d) or 30 days (Bt-30d) after inoculation. (**A**) Establishment of *B. thetaiotaomicron* in the gastrointestinal tract (GIT) of Bt-30d rats (n = 13) was monitored weekly by enumeration of the bacterial counts in the feces. (**B**) Scanning electron microscopy images of *B. thetaiotaomicron* in the cecum of Bt-2d and Bt-30d rats; scale bars, 1 μm. (**C**) Measurement of cecal pH in GF (n = 12), Bt-2d (n = 13) and Bt-30d rats (n = 19). (**D**) Cecal concentration of short-chain fatty acids (SCFA) in GF (n = 16), Bt-2d (n = 13) and Bt-30d rats (n = 19); only results for acetate, propionate and butyrate are shown, other SCFA were not detected; results are expressed in mM. Means with different letters are significantly different (*P*-value <0.05).

*B. thetaiotaomicron* colonized the GIT of recipient germfree (GF) rats quickly and stably: the maximum count, 10^10^ CFU/g of content, was attained with one day of inoculation and was maintained for at least 30 days (Figure 1A). Scanning electron microscopy of cecal contents of Bt-2d (2 days after inoculation) and Bt-30d (30 days after inoculation) rats revealed *B. thetaiotaomicron* as short bacilli with rounded ends (Figure 1B); the bacteria were associated with luminal contents, food-residue particles and shed mucus, consistent with previous descriptions in the distal gut intestine [20]. The cell surface of *B. thetaiotaomicron* differed between Bt-2d rats and Bt-30d rats. Most of the bacteria in Bt-2d rats but not those in Bt-30d rats exhibited granules on the cell surface (Figure 1B). The presence of *B. thetaiotaomicron* was associated with the cecal-content pH being much lower than that in controls (Figure 1C). Short-chain fatty acid (SCFA) production by *B. thetaiotaomicron* was investigated: acetate and propionate was produced within two days of implantation but no butyrate was produced (Figure 1D). Thus, two days after inoculation, *B. thetaiotaomicron* was stably and abundantly implanted and was metabolically active.

### *B. thetaiotaomicron* modulates the cell signaling pathway in the colon by favoring goblet cell differentiation

**Figure 2:**
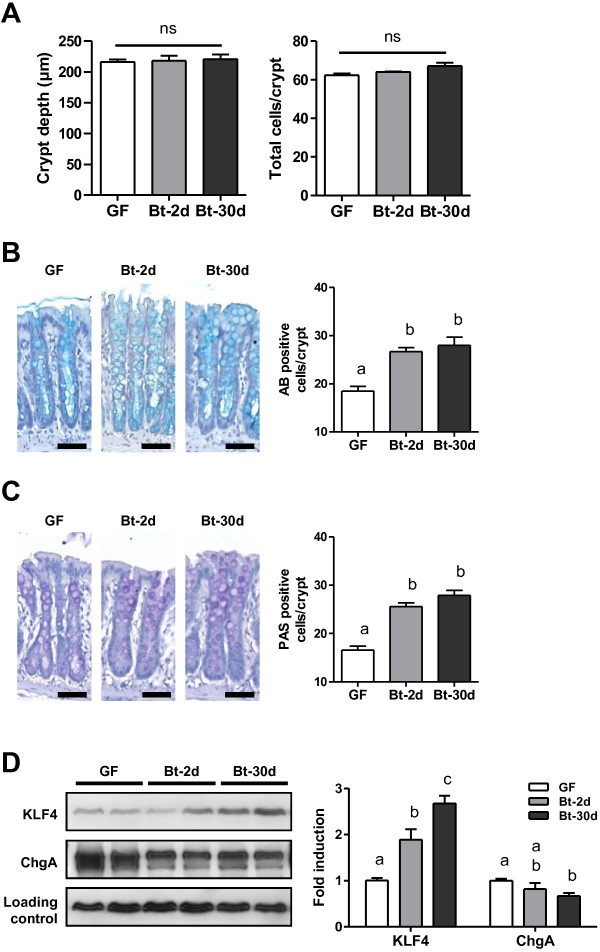
**Characterization of the colonic epithelial response in *B. thetaiotaomicron *mono-associated rats.** (**A**) Colonic crypt depth and total number of cells per crypt were determined on sections from GF (n = 3), Bt-2d (n = 5) and Bt-30d rats (n = 7) stained with Haematoxylin-Eosin-Safran (HES). (**B, C**) Representative pictures and graphs showing counts of goblet cells staining with (**B**) alcian blue (indicated as AB) and (**C**) periodic acid Schiff (indicated as PAS) in GF (n = 3), Bt-2d (n = 6) and Bt-30d (n = 7) samples. Scale bars, 50 μm. (**D**) Representative Western blots and densitometric analyses of proteins involved in the differentiation pathway of the secretory lineage, KLF4 and Chromogranin A (ChgA), in GF (n = 7), Bt-2d (n = 6) and Bt-30d (n = 7) samples. Means with different letters are significantly different (*P*-value <0.05).

Histological staining of the colonic epithelium from Bt-2d and Bt-30d mono-associated rats and from GF rats showed that there were no significant differences in general morphology, crypt depth or total numbers of cells per crypt (see Additional file 1: Figure S1A and Figure 2A). *B. thetaiotaomicron* is specialized in the degradation of polysaccharides, notably those of mucus. We, therefore, analyzed goblet cell counts and differentiation. The numbers of goblet cells stained either by Alcian blue (AB) or periodic-acid Schiff (PAS), specific for acidic and neutral mucopolysaccharides, respectively, were significantly higher in Bt-2d and Bt-30d rats than GF rats (Figure 2B, C). Similarly, the amount of KLF4 protein, a transcription factor involved in goblet cell terminal differentiation [30], was higher in Bt-2d and Bt-30d than GF rats (Figure 2D). Also, *muc2*, *muc4*, *c1galt1* and *b4galt4* mRNAs, encoding proteins involved in mucin synthesis and glycosylation, were significantly more abundant in Bt-2d and Bt-30d rats than in GF rats (Table 1). The *klf4* mRNA level was higher in Bt-2d, but not in Bt-30d, than in controls (Table 1). The amount of chromogranin A protein, a marker of enteroendocrine cells, another population of differentiated cells of the secretory lineage, was lower in Bt-30d rats than in control rats (Figure 2D). Thus, *B. thetaiotaomicron* appears to stimulate the goblet cell differentiation pathway of the secretory lineage.

**Table 1:** Analysis of host gene expression in the colonic epithelium

	**GF**	**Bt-2d**	**Bt-30d**	**Bt + Fp-30d**
***muc2***	1.04 ± 0.05 ^a^	1.52 ± 0.10 ^a,b^	1.71 ± 0.10 ^b^	1.79 ± 0.22 ^b^
***muc4***	1.00 ± 0.05 ^a^	3.51 ± 0.33 ^b^	2.69 ± 0.29 ^a,b^	2.83 ± 0.43 ^b^
***klf4***	1.01 ± 0.05 ^a^	1.30 ± 0.10 ^b^	1.09 ± 0.04 ^a,b^	1.08 ± 0.09 ^a,b^
***c1galt1***	1.00 ± 0.03 ^a^	2.28 ± 0.26 ^b^	1.23 ± 0.16 ^a^	1.54 ± 0.24 ^a,b^
***b4galt4***	1.02 ± 0.11 ^a^	3.89 ± 0.42 ^b^	2.13 ± 0.20 ^a,c^	3.00 ± 0.39 ^b,c^
***st3gal4***	1.05 ± 0.14 ^a^	2.98 ± 0.37 ^b^	1.39 ± 0.16 ^a^	1.67 ± 0.22 ^a^
***st6galnac3***	1.07 ± 0.27 ^a^	0.80 ± 0.29 ^a^	1,11 ± 0.55 ^a^	0.41 ± 0.11 ^a^
***gal3st3***	n.d.	n.d.	n.d.	n.d.
***chst7***	1.17 ± 0.36 ^a^	1.84 ± 0.55 ^a^	1.02 ± 0.23 ^a^	0.77 ± 0.20 ^a^

Relative abundance of *muc2, muc4, klf4*, *c1galt1, b4galt4*, *st3gal4, st6galnac3, gal3st3 and chst7* mRNAs in germfree (GF) (n = 4 to 10), Bt-2d (n = 4), Bt-30d (n = 6) and Bt + Fp-30d (n = 6). Means with different letters (a, b, c) are significantly different (*P*-value <0.05); n.d., not detected.

### Acetate, a bacterial metabolite produced by *B. thetaiotaomicron,* modulates goblet cells *in vitro*

**Figure 3:**
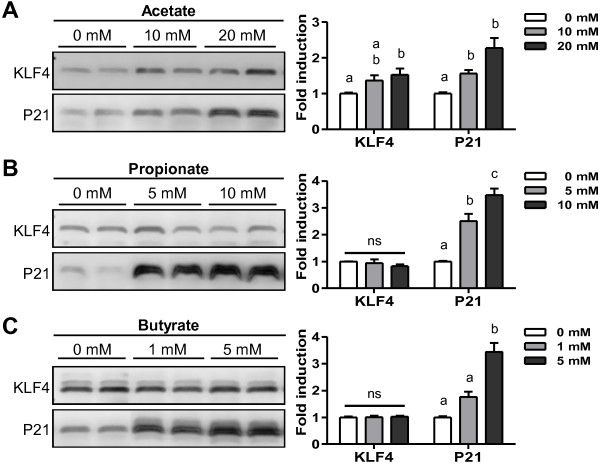
**Effects of bacterial metabolites on KLF4 protein in HT29-MTX cells.** Representative Western blot and densitometric analyses of KLF4 and P21 proteins in HT29-MTX cells incubated with (**A**) 0, 10, 20 mM acetate, (**B**) 0, 5, 10 mM propionate, and (**C**) 0, 1, 5 mM butyrate. Each graph reports means of three independent experiments with three internal repeats per experiment. Means with different letters are significantly different (*P*-value <0.05).

The main metabolites produced by *B. thetaiotaomicron* in the GIT were acetate (up to 6 mM) and propionate (up to 2 mM) (Figure 1D). We evaluated the effects of these bacterial metabolites on the amount of KLF4 protein produced by the mucus-producing cell line HT29-MTX [31]. The amount of KLF4 protein was higher in the presence than in the absence of acetate (10 and 20 mM) (Figure 3A) whereas propionate (5 and 10 mM) and butyrate (1 and 5 mM) had no effect (Figure 3B, C). The amount of butyrate-responsive protein, P21 [32], was increased by incubation of HT29-MTX cells with butyrate and there were similar trends with propionate and acetate (Figure 3). Thus, consistent with the findings *in vivo* in the presence of *B. thetaiotaomicron* (Figure 2), KLF4 protein production *in vitro* was up-regulated by acetate, the major metabolite produced by *B. thetaiotaomicron*. However, the stimulation was lower *in vitro* than *in vivo.*

### Metabolic cooperation between *B. thetaiotaomicron* and *Faecalibacterium prausnitzii in vitro* and *in vivo*

Bacterial metabolites are essential for epithelium homeostasis [33,34]. We investigated whether the effects of *B. thetaiotaomicron* are modulated in the presence of *F. prausnitzii* which is a major butyrate-producer and acetate-consumer [5].

**Figure 4:**
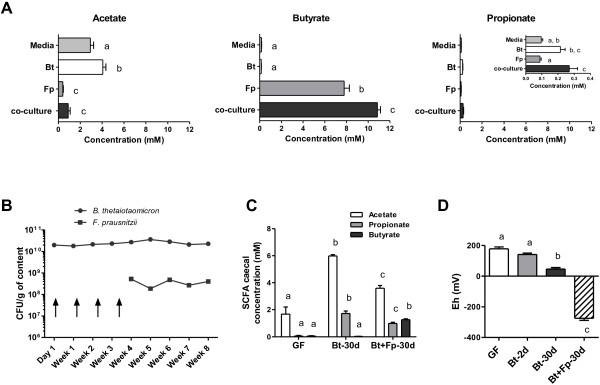
**Behavior of *B. thetaiotaomicron *and *F. prausnitzii *in combination *in vitro *and *in vivo*.** (**A**) Acetate, butyrate and propionate concentrations from culture media, pure cultures of *B. thetaiotaomicron* (Bt), *F. prausnitzii* (Fp) and cocultures. (**B**) Establishment of *B. thetaiotaomicron* and *F. prausnitzii* in the gastrointestinal tract (GIT) of Bt + Fp-30d rats (n = 16) was monitored weekly by enumeration in the feces; arrows represent the various inoculations with *F. prausnitzii* before successful colonization. (**C**) Cecal concentration of short chain fatty acids (SCFA) in the cecum of germfree (GF) (n = 12), Bt-30d rats (n = 19), and Bt + Fp-30d rats (n = 16). (**D**) Measurement of oxidoreduction potential (mV) in the cecal contents of GF (n = 11), Bt-2d (n = 13), Bt-30d (n = 19) and Bt + Fp-30d rats (n = 16). Means with different letters are significantly different (*P*-value <0.05).

We first checked *in vitro* that *B. thetaiotaomicron* produced acetate and propionate and that *F. prausnitzii* consumed acetate and produced butyrate (Figure 4A). When both bacteria were grown together, butyrate was produced rather than acetate. The butyrate production by *F. prausnitzii* was higher in the presence than absence of *B. thetaiotaomicron* (10.9 ± 0.3 mM versus 7.8 ± 0.5 mM) suggesting that *F. prausnitzii* synthesized butyrate from the acetate produced by *B. thetaiotaomicron*. We thus developed an experimental model of associated *B. thetaiotaomicron* and *F. prausnitzii* in rats (Figure 4B)*.* Two single consecutive inoculations of the two bacteria did not lead to a stable di-associated model. Inoculation with *F. prausnitzii* was thus repeated weekly (arrows on Figure 4B) but it became implanted only after week 4. From week 4 to week 8, a stable balance was maintained between the two bacteria, with *B. thetaiotaomicron* counts being 100-fold higher. In these Bt + Fp-30d rats, there was less acetate and more butyrate than in Bt-30d rats: 3.6 ± 0.2 mM acetate, 1.0 ± 0.1 mM propionate and 1.3 ± 0.1 mM butyrate in the cecal contents of Bt + Fp-30d rats, versus 6.0 ± 0.2 mM acetate, 1.8 ± 0.2 mM propionate and no butyrate in Bt-30d rats (Figure 4C). *F. prausnitzii* is an extremely oxygen-sensitive bacterium. We, therefore, monitored the Eh value, reflecting the oxido-reduction potential, in the cecum. The Eh value progressively decreased in the presence of *B. thetaiotaomicron* (141 ± 9 mV to 46 ± 10 mV for Bt-2d and Bt-30d, respectively) from the value found for GF rats (178 ± 13 mV) and decreased further in the presence of *F. prausnitzii*, that is, in Bt + Fp-30d rats (-275 ± 13 mV) (Figure 4D). Moreover, the pH of the cecal content in Bt + Fp-30d rats was significantly higher (6.00 ± 0.04) than that in Bt-30d rats (5.88 ± 0.01).

We were unable to obtain *F. prausnitzii* mono-associated rats since successful implantation of *F. prausnitzii* required the presence of *B. thetaiotaomicron*. In di-associated rats, *B. thetaiotaomicron* produced acetate and *F. prausnitzii* converted it into butyrate. We then used this gnotobiotic model, where both bacteria were metabolically active, to study the effects of these two commensal bacteria on the mucus production pathway.

### *F. prausnitzii* shifts the steady state induced by *B. thetaiotaomicron* in the colonic epithelium

**Figure 5:**
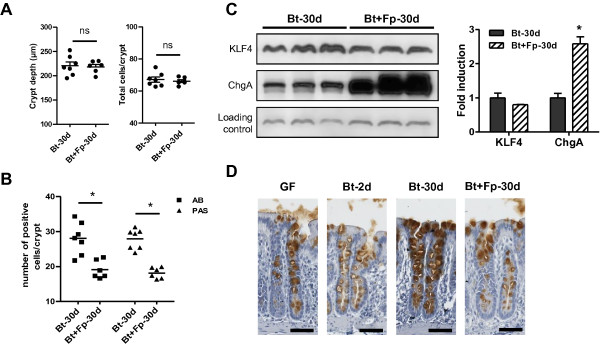
**Characterization of the colonic epithelial response in *B. thetaiotaomicron *and *F. prausnitzii *di-associated rats.** (**A**) Measurement of colonic crypt depth, total number of cells per colonic crypt and counts of (**B**) alcian blue- (indicated as AB) and periodic acid Schiff- (indicated as PAS) positive cells per crypt in colonic sections of Bt-30d (n = 7) and Bt + Fp-30d rats (n = 6). (**C**) Representative Western blot and densitometric analyses of proteins involved in the differentiation pathway of the secretory lineage, KLF4 and Chromogranin A (ChgA) in Bt-30d (n = 5) and Bt + Fp-30d rats (n = 4); protein fold induction in Bt-30d rats was used as a reference and arbitrarily defined as 1. (**D**) Immunostaining for MUC2 in germfree (GF) (n = 3), Bt-2d (n = 6), Bt-30d (n = 7) and Bt + Fp-30d rats (n = 6); scale bars, 50 μm. The asterisk indicates a statistical difference compared to Bt-30d rats (*P*-value <0.05); n.s., not significant.

In Bt + Fp-30d rats, colonic epithelium morphology, crypt depth and total number of cells per crypt were similar to those in Bt-30d rats (see Figure 5A and Additional file 1: Figure S1A). However, the numbers of AB- and PAS-positive cells per crypt were lower in Bt + Fp-30d than Bt-30d rats (see Figure 5B and Additional file 1: Figure S1B, C). Furthermore, the amount of KLF4 protein tended to be lower in Bt + Fp-30d than Bt-30d rats (Figure 5C). The amounts of *muc2*, *muc4*, *klf4*, *c1galt1* and *b4galt4* mRNAs were not different between Bt-30d and Bt + Fp-30d rats (Table 1). MUC2 staining was clearly lower in Bt + Fp-30d than Bt-30d rats and, indeed, was similar to that in GF rats (Figure 5D). The numbers of MUC2-positive cells tended to be lower in GF and Bt + Fp-30d rats (19.5 ± 0.6 and 21.6 ± 1.08 per crypt, respectively) than in Bt-30d rats (22.8 ± 1.7 per crypt). Chromogranin A protein was more abundant in Bt + Fp-30d than Bt-30d rats (Figure 5C). Thus, in this model with two metabolically complementary bacteria, *F. prausnitzii* seemed to attenuate the effects of *B. thetaiotaomicron* on goblet cell differentiation.

### Implantation of both commensal bacteria is associated with a shift in the mucin glycosylation profile

**Figure 6:**
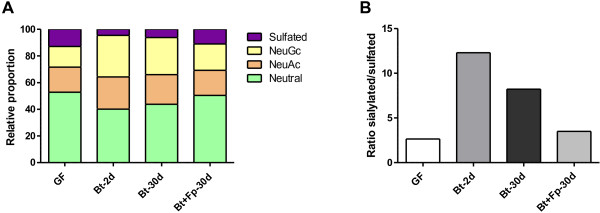
**Effects of bacterial colonization on colonic mucin glycosylation.** (**A**) Proportion of neutral and acidic oligosaccharides (carrying sulfate, N-acetyl neuraminic acid or N-glycolyl acid residues) in germfree (GF) rats (n = 3), Bt-2d (n = 2), Bt-30d (n = 3) and Bt + Fp 30d (n = 3) rats. (**B**) Ratio of sialylated to sulfated O-glycan chain in each group of rats.

Having investigated mucus quantity by evaluating goblet cell number and mucin synthesis, we assessed expression of genes encoding host enzymes and mucus quality by studying the profile of mucin O-glycosylation. We performed analyses of sialyltransferase (*st3gal4* and *st6galnac3*) and sulfotransferase (*gal3st3* and *chst7*) gene expression (Table 1). In Bt-2d rats, we observed an increase in the expression of the sialyltransferase s*t3gal4*. No modification was observed for the other genes tested. In parallel, we quantified the proportions of neutral/sulfated/sialylated oligosaccharides in the rat colonic mucins by studying the profile of mucin O-glycosylation. There were substantial changes to mucin glycosylation after colonization by *B. thetaiotaomicron* (see Additional file 2: Table S1). In Bt-2d rats, the proportions of sulfated (4.5% of total oligosaccharides instead of 12.9%) and neutral (40.1% instead of 52.8%) oligosaccharides were lower than in GF rats, and this was associated with higher proportions of O-glycans carrying NeuAc (24.2% instead of 18.9%) or NeuGc (31.2% instead of 15.4%) residues (Figure 6A). Only minor differences were observed between the mucins in Bt-2d and Bt-30d rats. The pattern of glycosylation of mucins from di-associated Bt + Fp-30d rats resembled that in GF rats. Interestingly*, B. thetaiotaomicron* increased the ratio of sialylated (NeuAc and NeuGc) to sulfated mucins in mono-associated compared to GF and di-asscociated rats (Figure 6B). Thus, as observed for goblet cell differentiation, *F. prausnitzii* seemed to attenuate the effects of *B. thetaiotaomicron*.

## Discussion

We report that commensal bacteria can influence goblet cells and mucin composition in the gut, providing new information about the relation among mucus, bacteria and intestinal homeostasis. *B. thetaiotaomicron* enhances goblet cell differentiation leading to an increase of goblet cell number and mucin gene expression in the colon of gnotobiotic rats. The presence of *B. thetaiotaomicron* also affects the composition of mucin O-glycans, with relative decreases in sulfated and neutral oligosaccharides in favor of sialylated oligosaccharides. *B. thetaiotaomicron*, therefore, appears to provoke modifications in the secretory lineage compared to GF rats, favoring mucus production and we could hypothesize that this is possibly for its own benefit. When *B. thetaiotaomicron* is associated with *F. prausnitzii*, which is an acetate consumer and a butyrate producer, the effects on both goblet cells and mucin O-glycosylation are attenuated. Using a novel gnotobiotic model, which is the first described involving *F. prausnitzii*, we showed that two major species of commensal bacteria can modulate the effects of the bacteria on goblet cells and thus on mucus production and mucin glycosylation.

The GIT is an environment with harsh constraints (pH, oxygen, access to nutrients) which shape the microbial composition. The genome of *Bacteroide*s species possesses gene clusters for capsular polysaccharide synthesis [17,18] and these polysaccharides are important for bacterial colonization of the GIT: they improve survival in the GIT, and also stimulate the immune system [35-37]. Scanning electron microscopy revealed differences in the *B. thetaiotaomicron* cell wall in Bt-2d and Bt-30d rats. Possibly, during the first days of adaptation to the GIT, *B. thetaiotaomicron* produce capsular polysaccharides which collapse through a dehydration process, as previously described [38,39]. The transcriptomic profile of *B. thetaiotaomicron* before and after its passage through GIT shows that *B. thetaiotaomicron* adapts its metabolism to survive in the GIT [20]. When *B. thetaiotaomicron* is associated with *Eubacterium rectale*, a representative of the *C. coccoides* group, both bacteria adapt their own metabolism to the presence of the other [6]. Thus, bacteria in the GIT presumably adapt to the host, and also to other bacteria [40]. In this study, we observed that in mono-associated rats, *B. thetaiotaomicron* induces goblet cell differentiation leading to an increase in mucin gene expression and goblet cell number, and a parallel decrease in chromogranine produced by enteroendocrine cell. These observations are in accordance with previous work showing that the colonic epithelium also adapts to the presence of bacteria [41-43]; these adaptations are parts of the mutual interactions essential for human health.

We show that, following colonization of the GIT by *B. thetaiotaomicron*, the pattern of mucin glycosylation changes, with increased expression of glycans carrying sialic acid residues (either NeuAc or NeuGc), correlated with decreased expression of sulfated and neutral oligosaccharides. A previous immunohistochemical study described similar alterations in the glycosylation pattern in the mouse intestine, induced by *B. thetaiotaomicron* implantation or by soluble factors produced by this microorganism [44]. *B. thetaiotaomicron* also induces fucosylation of intestinal epithelial cells, which is linked to its ability to use fucose, a component of glycosylated mucin [45]. Thus, glycan production or distribution is modulated by *B. thetaiotaomicron*, and this may involve processes important for signaling and mediating the host mucosal response. According to the enhancement of the expression of *st3gal4* in the presence of *B. thetaiotaomicron*, we can assume that there is a mechanism by which this bacterium modulates the composition of mucus chain O-glycans by impacting directly enzyme expression of the host. *B. thetaiotaomicron* may require high levels of expression of sialylated mucins for attachment and colonization of the GIT. Indeed, *B. thetaiotaomicron* may express adhesins specifically recognizing and binding to sialic acid residues and may also use sialic acid residues as host substrates. *B. thetaiotaomicron* possesses 28 predicted sulfatases and only 1 single predicted anaerobic sulfatase-maturating enzyme which allow bacteria to adapt to and forage on host sulfated glycans as nutrients [46]. Our observations of a substantial decrease in sulfated oligosaccharides in the rats colon two days after *B. thetaiotaomicron* colonization are consistent with this enzymatic potential. Presumably, *B. thetaiotaomicron* uses these sulfate residues rapidly not only as nutrient sources but also to gain access to other monosaccharides, such as N-acetylglucosamine (GlcNAc) and galactose (Gal), the two monosaccharides substituted by sulfate residues on mucin O-glycans. In a favorable ecosystem (that is, in the absence of other microorganisms), there is probably no need for the bacteria or the host to maintain a high level of expression of sulfated glycans. However, when *B. thetaiotaomicron* is associated with *F. prausnitzii*, the proportion of sulfated glycans is greater than that observed in the presence of *B. thetaiotaomicron* alone. This suggests that, in a more complex environment involving a competitive ecosystem, sulfated glycans may confer an ecological advantage on *B. thetaiotaomicron*.

We were unable to obtain, on a large scale and reproducibly, *F. prausnitzii* mono-associated rats; the implantation of *F. prausnitzii* required the prior presence of *B. thetaiotaomicron*. Furthermore, *F. prausnitzii* was unable to colonize the GIT early after the introduction of *B. thetaiotaomicron*. This may have been due to the physicochemical environment. *F. prausnitzii* became established in the gut only after a decrease in the oxidoreduction potential, caused by the presence of *B. thetaiotaomicron*, suggesting that *B. thetaiotaomicron* “prepares” the GIT to accommodate sustainably more oxygen-sensitive bacteria. *F. prausnitzii* and, to a larger extent, the *C. leptum* group, arrive in the GIT late after birth, presumably because they are sensitive to oxygen. Moreover, after intestinal resection, disrupting the gut structure and exposing the colon to oxygen, the *C. leptum* group disappears from the microbiota [47]. In the case of strict anaerobes like *F. prausnitzii*, their ability to colonize is governed by the physicochemical constraints of gut rather than by any lack of metabolic substrates. This contrasts with *Lactobacillus bulgaricus*, which is an aerobic-tolerant lactic acid bacterium, and which does not colonize the gut of GF rats in the absence of lactose [48]. That colonization by *B. thetaiotaomicron* precedes the implantation of *F. prausnitzii* gives clues about mechanisms governing microbial ecology and processes of colonization.

The two bacteria were metabolically complementary both *in vitro* and *in vivo*. *F. prausnitzii* consumes acetate produced by *B. thetaiotaomicron* and in turn produces butyrate. Short-chain fatty acids (SCFA) trigger pleiotropic signals in the host, including signals regulating mucin synthesis and secretion [33,49-51]. SCFA, in particular butyrate and to a lesser extent acetate, are reported to have inducer effects on mucin synthesis and production *in vitro*[33,49,51]. Butyrate also increases the expression of the transcription factor *klf4* in HT29 cells [52]. On the contrary, another experiment reported an inhibitor effect of butyrate on mucin synthesis *in vitro*[53]. All these data show the potential role of SCFA in modulating the mucus pathway and it may be a mechanism by which they affect the goblet cell differentiation pathway in our gnotobiotic models. Using HT29-MTX, a mucus producing cell line, we showed that acetate increases KLF4 abundance. Understanding the effects of the microbiota on mucus production and goblet cell differentiation is of major importance as mucus acts as a protective barrier and disruption of this mucus layer leads to inflammation [54]; abnormalities of the mucus layer, and modulations of goblet cells and mucin secretion have been described in inflammatory bowel diseases [55]. In a murine model of DSS-induced colitis, the mucus layer is altered before the induction of any inflammation evidencing the importance of mucus layer integrity to prevent inflammation [56]. The O-glycosylation pattern of mucins has also a determinant role in health, with sulfated colonic mucins playing a protective role [57]. Indeed, mucins are poorly sulfated in patients with ulcerative colitis [58]. Butyrate induces the expression of sulfotransferases, the enzymes catalyzing sulfatation of mucin in the mouse colon [57] and also up-regulates expression of galactose-3-O-sulfotransferase 4 in human intestinal epithelial goblet cells [59]. The increase in sulfated mucins observed in our *B. thetaiotaomicron* and *F. prausnitzii* di-associated rats is consistent with these previous studies. We, therefore, suggest that metabolites, mainly acetate and butyrate, produced by *B. thetaiotaomicron* and *F. prausnitzii*, respectively, may be responsible, at least in part, for the modifications that we observed in goblet cells and mucin O-glycosylation.

## Conclusions

Our study reveals relationships between intestinal microbiota and the differentiation and secretory program of colonic goblet cells. We propose that *B. thetaiotaomicron* and *F. prausnitzii*, two main members of microbiota representative of Bacteroidetes and Firmicutes, contribute to the establishment of epithelial homeostasis. These two bacteria, which are metabolically complementary, modulate *in vivo* the intestinal mucus barrier by modifying goblet cells and mucin glycosylation. Here we show how the balance between *B. thetaiotaomicron and F. prausnitzii* could be determinant for maintaining colonic epithelial homeostasis and health *via* their respective effects on mucus.

## Methods

### Bacterial strains and culture conditions

The reference strains *B. thetaiotaomicron* VPI-5482 (ATCC 29148) and *F. prausnitzii* A2-165 (DSM 17677), isolated from human fecal stool, were used. Bacteria were grown overnight at 37°C in YBHI medium (brain-heart infusion medium supplemented with 0.5% Difco yeast extract (Becton Dickinson and Company, Le Pont De Claix, France) supplemented with 1 mg/ml cellobiose, 1 mg/ml maltose and 0.5 mg/ml L-cysteine (all from Sigma-Aldrich, St-Louis, MO, USA) in an anaerobic chamber containing a gas mix of 90% N_2_, 5% CO_2_ and 5% H_2_. For co-culture, the 24 h pre-culture of each was added to fresh medium at 1/10 and 1/100 for *F. prausnitzii* and *B. thetaiotaomicron*, respectively.

*B. thetaiotaomicron* and/or *F. prausnitzii* were enumerated weekly in the feces of mono- and di-associated rats. Fresh feces were introduced into the anaerobic chamber, serially diluted 10-fold and plated on supplemented YBHI agar. To enumerate only *F. prausnitzii* in the feces of di-associated rats, 1 ml of the 10^-2^ dilution of the feces was treated with 0.1 ml of sodium azide (3%) for five minutes at 37°C and then serially diluted 10-fold and plated on supplemented YBHI agar with added sodium azide (0.02%).

### Animals and experimental design

All procedures were carried out according to European and French guidelines for the care and use of laboratory animals (permission 78–123, granted to M.T.). GF rats (males, Fisher 344) were used. Animals were born and bred at the Institut National de la Recherche Agronomique (Jouy-en-Josas, France). All rats were housed in Trexler-type isolators (La Calhène, Vélizy, France) and fed with a standard diet, rich in polysaccharides (R03, SAFE) sterilized by gamma irradiation (45 kGy). GF rats were used as controls (n = 16). To obtain rats mono-associated with *B. thetaiotaomicron*, GF rats were inoculated by a single oral gavage with 1 ml of culture of *B. thetaiotaomicron* (10^7^ CFU/ml) and euthanized 2 days (Bt-2d rats, n = 13) or 30 days (Bt-30d rats, n = 19) after inoculation. To obtain rats di-associated with *B. thetaiotaomicron* and *F. prausnitzii*, GF rats were orally inoculated with 1 ml of culture of *B. thetaiotaomicron*; 24 h later, when this bacterium was established in the GIT, 1 ml of 100× concentrated culture of *F. prausnitzii* (7.10^9^ CFU/ml) was administered orally to a donor rat pretreated with 0.1 ml of sodium bicarbonate (0.2 M). This manipulation was repeated every week until *F. prausnitzii* was established in a donor rat. Once one rat di-associated with the two bacteria was obtained, its feces were administrated orally to additional rats pretreated with sodium bicarbonate. Rats were euthanized 30 days after *F. prausnitzii* implantation (Bt + Fp-30d rats, n = 16).

All rats were euthanized at the age of three months; rats were anesthetized at 9:00 a.m. with isoflurane, cecal contents were collected and colons were recovered and immediately used for cell isolation or histology.

### Scanning electron microscopy

Scanning electron microscopy [60] analyses were performed on the MIMA2 platform (INRA, Massy, France). Aliquots of 0.2 g of cecal contents were suspended and fixed in 200 μl of glutaraldehyde, 3% ruthenium red for two hours in an anaerobic chamber and then stored at 4°C. Scanning electron microscopy was performed as reported previously [47].

### Short chain fatty acid analysis

Acetate, propionate and butyrate concentrations were determined in cecal contents, supernatants from bacterial culture and culture media after water extraction of acidified samples using gas liquid chromatography (Nelson 1020, Perkin-Elmer, St Quentin en Yvelines, France) as described previously [61]. SCFA concentrations are expressed in mM.

### Oxidoreduction potential (Eh) and pH measurement

The measurement of pH and oxidoreduction potential was as described by Martin *et al*., after a total immersion of the electrode in the cecal content [62]. A combined autoclavable electrode (InLAb®, Mettler-Toledo SAS, Viroflay, France) was used for all measurements.

### Histology and immunohistochemisty assays

Flushed colons were opened longitudinally and cut into 2 cm sections. The samples were fixed in 4% paraformaldehyde (4 h, room temperature), dehydrated, and embedded in paraffin according to standard histological protocols. Four-micrometer thick sections of distal colon were mounted on SuperFrost® Plus slides (Thermo Fisher, Waltham, MA, USA). Paraffin-embedded sections were deparaffinized and stained with: Haematoxylin-Eosin-Safran (HES) to measure crypt depth and Alcian blue (AB) solution pH 2.5 or periodic acid-Schiff (PAS) reagent to count goblet cell number per crypt. MUC2 immunostaining was performed using the EnVision + System-HRP (Dako-Cytomation, Trappes, France) and anti-MUC2 (1:5,000; sc-15334, Santa Cruz Biotechnology, Heidelberg, Germany) as the primary antibody as previously described [12]. For all markers studied (HES, AB, PAS, MUC2), only U-shaped longitudinally cut crypts with open lumina were considered. Results reported are means obtained by analysis two distal colon sites for each rat and at least 10 crypts per site with NanozoomerDigitalPathology view software (Hamamatsu Photonics, Hamamatsu, Japan).

### Western blot analysis

Colonic epithelial cells were isolated from the whole colon according to the method described by Cherbuy *et al.*[63]. The cell pellet from whole colon or HT29-MTX cell cultures was immediately used for protein extraction [41] and Lowry’s procedure was used for protein assays [64]. Western blot analysis was performed as previously described [41] using 12% SDS-PAGE and anti-KLF4 (1:500; IMG-6081A, Imgenex, San Diego, CA, USA), anti-Chromogranin A (1:500; ab15160, Abcam, Cambridge, MA, USA), or anti-P21 (1:200; sc-397, Santa Cruz Biotechnology) with appropriate peroxidase-conjugated secondary antibodies (Jackson ImmunoResearch Laboratories, West Grove, PA, USA). For each Western blot, protein loads were determined using anti-cullin1 antibodies (1:400; sc-17775, Santa Cruz Biotechnology). Signals detected on autoradiographic films were quantified by scanning densitometry with Biovision 1000 and Bio-1D software (Vilber Lourmat, France).

### Total RNA extraction and real-time quantitative PCR analysis

Total RNA was extracted from isolated colonic epithelial cells by the guanidinium thiocyanate method [65]. RNA concentration and purity were determined by absorbance measurement using a nanodrop ND-1000 (Thermo Fisher Scientific, Illkirch, France) and RNA Integrity Number (RIN) was checked using the RNA 6000 Nano LabChip® kit (Agilent Technologies, Santa Clara, CA, USA) and the Agilent 2100 bioanalyzer at ICE platform (INRA, Jouy-en-Josas, France). All RNA had a RIN between 8 and 10, indicating a high RNA quality in all samples. Reverse transcription was performed with 7 μg of RNA of each sample using the High-Capacity cDNA Archive Kit (Applied Biosystems by Life Technologies SAS, Saint Aubin, France) according to the manufacturer’s instructions. Each cDNA sample was tested for PCR inhibition with the TaqMan® Exogenous Internal Positive Control (Applied Biosystems) and no inhibition was detected for any sample. The cDNA products were analyzed in triplicate by RT-qPCR with an ABI PRISM 7000 Sequence Detection System and the 7000 system software version 1.2.3 (Applied Biosystems). Several genes were analyzed using TaqMan Gene Expression Assays (Applied Biosystems): *klf4* (Rn00821506_g1), *muc2* (Rn01498197_m1), *muc4* (Rn01475265_m1), *c1galt1* (Rn01455817_m1), *b4galt4* (Rn01439430503812_m1), *st3gal4* (Rn01786289_m1), *st6galnac3* (Rn00569406_m1), *gal3st3* (Rn01233548_m1) and *chst7* (Rn01430599_m1). 18S rRNA (Hs99999901_s1) was used as a reference. Results obtained were normalized to the value for 18S rRNA and compared with the mean target gene expression in GF rats as the calibrator sample. The following formula was used: fold change = 2^-ΔΔCt^, where ΔΔCt threshold cycle (Ct) equals (target Ct - reference Ct) of sample minus (target Ct - reference Ct) of the calibrator.

### Cell culture

The mucus-secreting cell line HT29-MTX was obtained from Thecla Lesuffleur (INSERM UMR S 938, Paris, France) and used from passages 27 to 36 [31]. Cells were routinely grown in Dulbecco’s modified Eagle’s minimal essential medium (DMEM) with 4.5 g/L glucose (Lonza, Verviers, Belgium), supplemented with 10% (v/v) fetal calf serum (FCS) inactivated by incubation for one hour at 56°C (Lonza, Verviers, Belgium), with 1% (v/v) L-Glutamine 200 mM (Lonza). The experimental design involved cells being grown for seven days in six-well tissue culture plates at a density of 12.10^4^ cells per ml. Cells were maintained at 37°C in a 10% CO_2_:90% air atmosphere. The culture medium was changed daily. Undifferentiated cells were obtained and incubated with 0, 10, 20 mM acetate or 0, 5, 10 mM propionate or 0, 1, 5 mM butyrate for 17 h. To avoid a pH effect, when SCFA were added in culture medium, the pH value was checked and adjusted to those of culture medium. Each condition was tested in triplicate and experiments were repeated three times. Cells were scraped off and immediately used for protein extraction.

### Analysis of mucin glycosylation

Rat colonic mucosa was scraped and mucins were purified as previously described [66]. Briefly, mucins were solubilized in 4 M guanidine chloride solution containing 5 mM EDTA, 10 mM benzamidine, 5 mM N-ethylmaleimide, 0.1 mg/mL soy bean trypsin inhibitor and 1 mM PMSF (phenylmethanesulfonyl fluoride). Bacteria were removed by centrifugation at 3,000 g for 15 minutes. Supernatant was collected and CsCl was added to give a density of 1.4 g/mL and mucins were purified by isopycnic density-gradient centrifugation (Beckman Coulter LE80K ultracentrifuge; 70.1 Ti rotor, 58,000 rpm at 15°C for 72 h; Beckman Coulter France S.A.S, Villepinte, France). The mucin-containing fractions were pooled, dialyzed against water and lyophilized. Mucins were then subjected to β-elimination under reducing conditions (0.1 M NaOH, 1 M KBH4 for 24 h at 45°C). The mixtures of oligosaccharide alditols were permethylated by the sodium hydroxide procedure. After derivation, the reaction products were dissolved in 200 μl of methanol and further purified on a C18 Sep-Pak (Waters Corporation, Milford, MA, USA). Permethylated oligosaccharides were analyzed by MALDI TOF mass spectrometry in positive ion reflective mode as [M + Na]+. The relative proportions of each oligosaccharide were calculated by integration of peaks on MS spectra, and are expressed as percentages of the total.

### Statistical analyses

Data are reported as means ± SEM for the number of animals indicated. Group data were compared using one-way analysis of variance (ANOVA) followed by a Wilcoxon test (jmp version 7) when the ANOVA revealed differences between groups. Differences with *P*-value <0.05 were considered to be statistically significant.

## Abbreviations

AB: Alcian blue; Ct: Threshold cycle; DMEM: Dulbecco’s modified Eagle’s minimal essential medium; FCS: Fetal calf serum; Gal: galactose; GF: germfree; GIT: Gastrointestinal tract; GlcNAc: N-acetylglucosamine; HES: Haematoxylin-Eosin-Safran; PAS: Periodic-acid Schiff; RIN: RNA Integrity Number; SCFA: Short-chain fatty acid.

## Competing interests

The authors have no conflicting financial interests.

## Authors’ contributions

LW, SM, CC, PL and MT designed *in vitro* and *in vivo* experiments and LW, SM, MLN, SB, MJCC, VR, CP, CB and CRM performed these experiments. LW, SM, CRM, PL and MT wrote the manuscript. All authors read and approved the final manuscript.

## Supplementary Material

Additional file 1: Figure S1 Characterization of the colonic epithelial response in *B. thetaiotaomicron* and *F. prausnitzii* di-associated rats. (**A**) Representative pictures of colonic sections stained with HES in GF, Bt-2d, Bt-30d and Bt + Fp-30d rats. Representative pictures showing goblet cells staining with (**B**) alcian blue (indicated as AB) and (**C**) periodic acid Schiff (indicated as PAS) in Bt-30d rats and Bt + Fp 30d (n = 3) rats. Scale bars, 50 μm. Click here for file

Additional file 2: Table S1 Deduced monosaccharide composition of mucin O-glycans from GF, Bt-2d, Bt-30d and Bt + Fp 30d rats, as identified by MALDI-TOF mass spectrometry in positive ion mode. The relative proportions of each oligosaccharide were calculated by integration of peaks on MS spectra. Mucus O-glycans types were separated into neutral oligosaccharides (blue), oligosaccharides carrying sialic acid (orange) and sulfated residues (green). Results are presented as means ± SD of the percentage of each oligosaccharide. Click here for file
