# Supplementary figures and images for: Bacteroides thetaiotaomicron and Faecalibacterium prausnitzii influence the production of mucus glycans and the development of goblet cells in the colonic epithelium of a gnotobiotic model rodent
Source: BMC Biol. 2013 May 21;11:61. doi: 10.1186/1741-7007-11-61 (PMC3673873; doi:10.1186/1741-7007-11-61)

## Slide 1
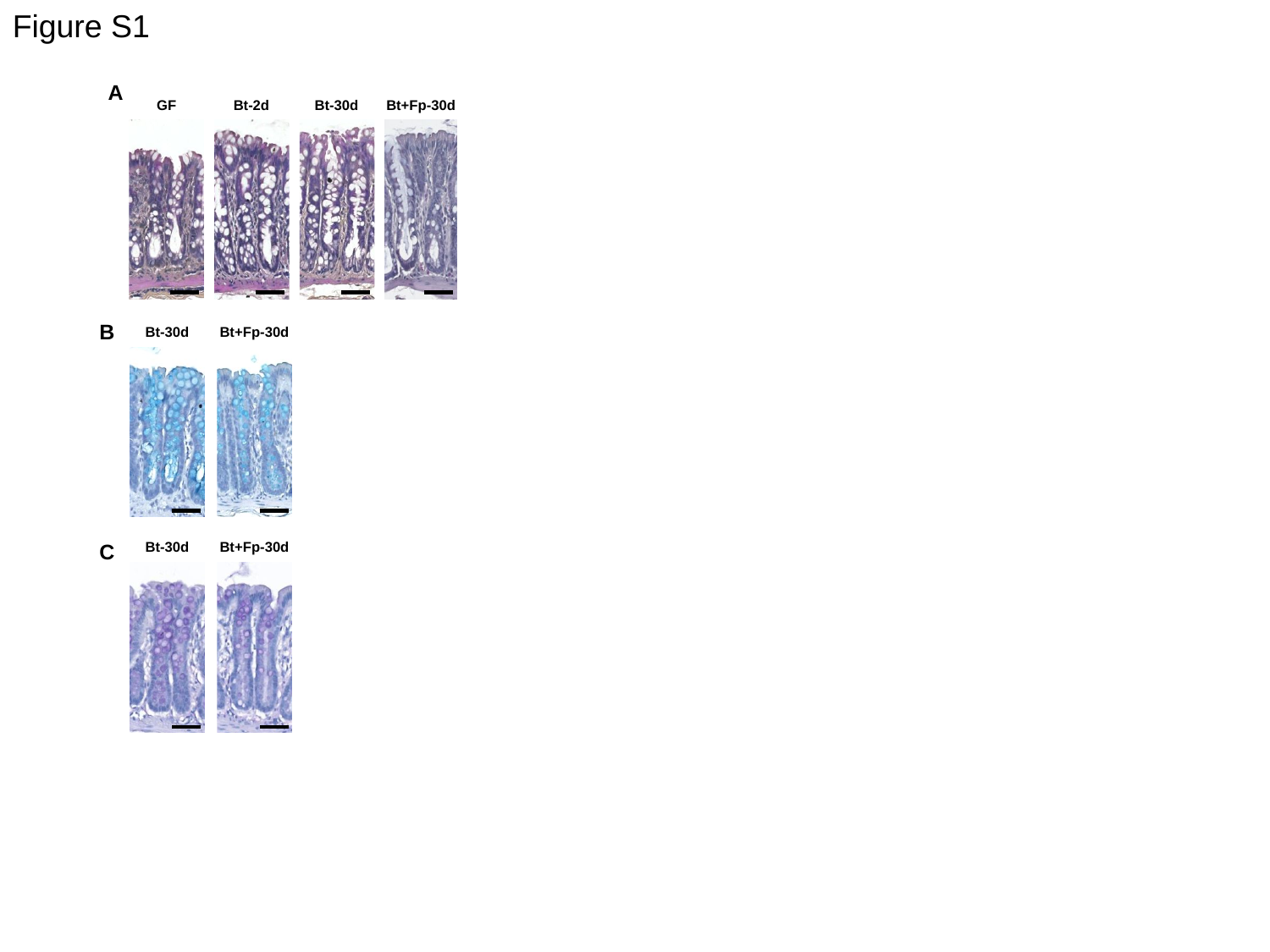

Figure S1
A
GF
Bt-2d
Bt-30d
Bt+Fp-30d
B
Bt-30d
Bt+Fp-30d
Bt-30d
Bt+Fp-30d
C

Supplement: Additional file 1: Figure S1 — Characterization of the colonic epithelial response in B. thetaiotaomicron and F. prausnitzii di-associated rats. (A) Representative pictures of colonic sections stained with HES in GF, Bt-2d, Bt-30d and Bt + Fp-30d rats. Representative pictures showing goblet cells staining with (B) alcian blue (indicated as AB) and (C) periodic acid Schiff (indicated as PAS) in Bt-30d rats and Bt + Fp 30d (n = 3) rats. Scale bars, 50 μm. [file 1741-7007-11-61-S1.pptx]
